# An Approach to Measuring Dispersed Families with a Particular Focus on Children ‘Left Behind’ by Migrant Parents: Findings from Rural South Africa

**DOI:** 10.1002/psp.1843

**Published:** 2014-01-08

**Authors:** Rachel Bennett, Victoria Hosegood, Marie-Louise Newell, Nuala McGrath

**Affiliations:** 1 University of SouthamptonSouthampton, UK; 2 Africa Centre for Health and Population Studies, University of KwaZulu-NatalKwaZulu-Natal, South Africa

**Keywords:** left behind children, migrant parents, dispersed families, migration measurement issues, South Africa

## Abstract

There is growing policy and academic interest in the conditions, experiences, and well-being of migrant families stretched across origin and destination households. In South Africa, the dispersal of children and migrant parents across multiple households is a commonplace childhood experience. However, in common with the broader international context, quantitative analyses of the social and residential connections between children and migrant parents in South Africa have been limited by the lack of available data that document family arrangements from the perspective of more than one household. This paper describes a new data collection effort in the origin and destination households of migrants from rural KwaZulu-Natal and explains the methodology for using this data to examine multiple household contexts for children and parents. In order to illustrate the contribution that this form of data collection effort could make to family migration studies, the paper also presents results on the living arrangements of children ‘left behind’ by migrant parents; a potentially vulnerable group whose arrangements are challenging to examine with existing data sources. The empirical results show the majority (75%) of left behind children have previously migrated and a significant proportion of migrants' children (25%) were not living in their parent's origin or destination household. The findings highlight the need for careful measurement of the circumstances of left behind children and demonstrate the contribution of linked data for providing insights into the residential arrangements of migrants' children. © 2014 The Authors. *Population, Space and Place* published by John Wiley & Sons Ltd

## Introduction

An emerging family migration literature has documented the experiences and conditions of children ‘left behind’ when one or both parent(s) migrate and the resulting forms of trans-local family (DeWind & Holdaway, 2005; Whitehead & Hashim, 2005; Toyota *et al*., 2007; Yeoh & Lam, 2007). In Southern Africa, qualitative studies of dispersed family arrangements in the context of very high levels of adult migration provide insights into the ways in which parent–child relationships are operationalised (Murray, 1980; Jones, 1993; Spiegel *et al*., 1996; Townsend, 1997; Madhavan *et al*., 2008). However, in common with many other high-migration contexts, there is a lack of quantitative data available with detailed information about the interconnectedness and differences in the social and residential arrangements of children and migrant parents. This is in part because of the conceptual and methodological challenges associated with capturing quantitative data on family relationships between people living in different places. Conceptualised and operationalised using a sampling frame of households' resident in the study area, household surveys and censuses frequently do not collect information about residentially or socially dispersed family or household members. Particularly scarce are detailed data about the linkages and exchanges between the origin household and the residential locations of household members living elsewhere. In this paper, we use surveillance data from the Africa Centre Demographic Information System (ACDIS) in rural KwaZulu-Natal, South Africa, and a new nested sample survey of migrants conducted in their destination households, the Non-Residents Living Arrangements (NRLA) survey. The objectives of this paper are (i) to describe the methodology for using data collected in multiple households for examining the social and residential connections between children and migrant parents and (ii) to illustrate the contribution combining data from migrants' origin and destination households can make to family demography, by examining the circumstances of one group of potentially vulnerable children, those left behind by migrant parents. The paper begins with an overview of family migration in South Africa, particularly the circumstances of left behind children, and the data challenges associated with examining dispersed family relationships in the context of migration. The subsequent sections describe the data and methodological techniques and present empirical findings on the residential arrangements of left behind children. The final section draws conclusions about the utility of linked data from multiple households for family demography, with a particular focus on understanding the circumstances of left behind children.

### The context of family migration in South Africa

Circular adult labour migration has been deeply entrenched in South Africa's social and economic systems since the early 20th century. During the colonial and apartheid eras, permanent family migration amongst non-white population groups was inhibited by legislation designed to control settlement in urban areas (Jones, 1993; Moser, 1999). Consequently, migrants, most often but not exclusively male, would frequently ‘leave’ their families for periods of time in order to gain employment and contribute to the resources of their origin households. In contemporary rural South African communities, levels of temporary adult migration remain high (Collinson, 2009; Muhwava *et al*., 2010; Reed, 2013). Despite the removal of restrictions on family migration, and the growth of women's participation in migration, studies suggest that most parents continue to migrate without bringing their children to live with them in their destination household (Posel, 2010). Kautzky's (2009) analyses of migrant parents' choices in the Agincourt, subdistrict of the Mpumalnga Province, indicated that 89% of parents did not move at least one child with them.

### Focus on left behind children

Where rates of unaccompanied parental migration are very high, as is the situation in rural communities in South Africa, there has been interest in identifying the impact of parental migration on children's well-being. Labour migration is a means by which adults can secure resources that can be used to promote the health and opportunities of their children. However, the types of residential and social instability that can be associated with parental migration may place children at risk of reduced well-being. As Cooke (2008) and Root and De Jong (1991) note in international commentaries on family migration, the circumstances under which children are ‘left-behind’ when one or both parents migrate may vary widely and are likely to be complex and dynamic. Left behind children are often reliant on extended kin for care (Orellana *et al*., 2001) and where families are spatially dispersed, this implies movement on the part of the child. In the South African context, Ford and Hosegood (2005), analysed longitudinal population-based data from rural KwaZulu-Natal and found that children with mothers who migrated during a one year period were 42 times more likely to migrate in the observation window than other children (95% CI: 36.4, 48.4), although they were not able to establish whether children accompanied their migrant mother or migrated elsewhere. Though a major reason, parental migration is not the only reason for the residential separation of children and their parents in South Africa. Extra-marital childbearing, union instability, orphaning, as well as the high level of independent migration of children themselves motivated by education and care needs, are all common contributing factors (Russell, 2003; Hosegood *et al*., 2009). Children who are not resident in their migrant parent's origin household when the parent migrates may have a different experience of parental migration to those that were co-resident with a parent. Recent calls to support families to promote the health and wellbeing of children in South Africa have drawn attention to the need to better understand the spatial distribution of children in relation to parents and family members (Sherr *et al*., 2008; Hosegood & Madhavan, 2010; Hall & Posel, 2012).

### Measuring dispersed family relationships in the context of adult migration

Data sources for countries with high levels of mobility, including South Africa, are now moving towards a non-residential definition of household membership. This makes it possible to identify the origin households of migrant parents and children if they share social membership of the same household. Studies which have examined the well-being of children left behind frequently focus on children who are resident members of a household with one or more non-resident members (e.g. Lu & Treiman, 2007; Collinson, 2009). However, it remains problematic to identify migrants' children who do not share household membership(s) with their migrant parent(s). For example, children with migrant fathers who live with maternal kin are unlikely to share household membership with their father.

Few sources of available data include information on links to other households, such as the destination household of migrants. This limits the scope of analyses that focus on migrants' children in several ways. Firstly, it is not possible to consider the circumstances and living arrangements of migrant parents who do and do not have children living with them in their destination household or to compare the characteristics and well-being of children in different arrangements. Secondly, it is not possible to tell whether migrant parents and children follow common migratory routes. Children may live in a different household as a result of parental migration, for example, with extended family. Hence, migrant parents and children from the same origin household may not have the same destination household. In this scenario, it is possible that the child's migrant parent will not be listed on the household roster of the child's destination household, particularly if the parent has never been a resident with that household. Such children are therefore unlikely to be classified as children of migrants but nonetheless are effectively children who have been left behind in the sense that they are not co-resident with their migrant parent in his or her destination household.

A small number of studies have used a matched sample methodology to collect data from origin and destination households of migrants in order to be able to consider both simultaneously. However, these studies are typically based on small non-representative samples. For example, one study interested in the family relationships of Ghanaian migrants sought to identify migrants in Amsterdam and family members in Ghana (Mazzucato, 2008). There was no baseline survey of migrants in their destination or origin communities; thus, the sampling strategy was based on chance encounters and contact with community organisations, and the sample size achieved was moderate. In this paper, we discuss and evaluate a new approach to measuring dispersed family relationships, which combines detailed longitudinal data on origin households from a Demographic Surveillance System (DSS) with a nested sample survey conducted in the destination households of adult migrants. The DSS data provides a sampling frame for migrants and a source of information about the origin household and previous child and adult migration, and the survey data provides information on the composition of migrants' destination households.

### Data sources and methods

**Figure 1:**
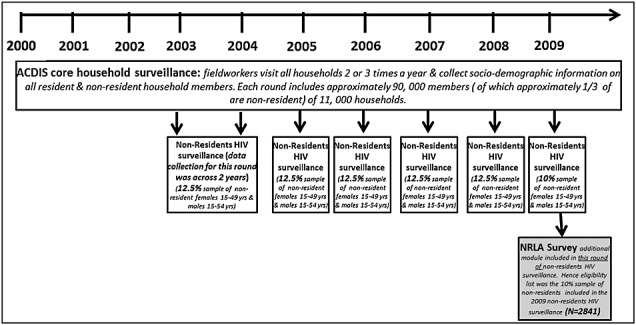
Relationship between the Africa Centre Demographic Information System, non-residents HIV surveillance, and Non-Residents Living Arrangements survey.

This study is based on data from the Non-Residents Living Arrangements (NRLA) survey, a cross-sectional survey of non-resident members of rural households included in the Africa Centre Demographic Information System (ACDIS) who were contacted as part of a tracking exercise for non-residents HIV surveillance. Figure 1 summarises the relationship between the data sources. Data collection for the NRLA survey, non-residents HIV surveillance, and the ACDIS are conducted under the auspices of the Africa Centre for Health and Population Studies. Ethical approval for data collection is granted by the University of KwaZulu-Natal Nelson Mandela medical school. The following sections provide a detailed description of the data sources and methods for linking the data to examine the social and residential connections between children and parents.

#### Africa Centre Demographic Information System

The ACDIS has been in operation since 2000 and contains detailed socio-demographic data about the population of a predominately rural 438 km^2^ demographic surveillance area (DSA) in northern KwaZulu-Natal (Tanser *et al*., 2008). Each round of data collection includes approximately 90,000 members of the 11,000 households resident in the study area (Tanser *et al*., 2008). The average household size is 7.9 members and the primary sources of income for most households are state pensions and/or waged employment.

The principle of DSSs is to maintain a dynamic list of all individuals living within a geographically defined area. The list records who belongs to households in the area, when events such as births, deaths, and migratons occur, and with whom each individual is living with or connected to (Tollman, 2008). The ACDIS was designed with special attention to the definitions of households and social relationships, in order to reflect the complexity and fluidity of living arrangements in South Africa (Hosegood & Timaeus, 2005). There are three main subjects of interest that are observed longitudinally: (i) bounded structures (homesteads); (ii) households; and (iii) individuals. All registered bounded structures in the surveillance area are visited by fieldworkers, and information is collected about the households who are resident there. A household is a social group with one or more members. Household membership is respondent-defined and primarily relates to perceptions of social connectedness and belonging. Individuals are eligible for inclusion in the ACDIS if they are considered a member of a household in the surveillance area.

Although individual circumstances can vary widely, a person's place of residence will be broadly related to the place where they currently usually sleep and keep their belongings. As household membership is not conditional on residency, an individual can be recorded as a non-resident household member if they are residing in a household outside the DSA but remain socially connected to a household in the DSA. Changes in residence by individuals and households are identified within the DSA (internal migration) and into or out of the DSA (external migration). In mid 2009, 37% of adults and 18% of children (<18 years) were non-resident household members living outside the DSA.

Longitudinal follow-up of all individuals and households is conducted during routine household visits by fieldworkers two (until 2012) or three (since 2012) times a year. Participation rates for household surveillance are >99% (Tanser *et al*., 2008). Information is collected on all resident and non-resident household members and includes data on births, deaths, migrations, marriages, and parental survival. Household socio-economic surveys have been conducted annually with the exception of 2000, 2002, 2004 and 2008, and collect information about the socio-economic characteristics of households (e.g. asset ownership) and household members (e.g. educational attainment and employment status).

#### Non-residents HIV surveillance

In addition to the routine household surveillance, the Africa Centre also conducts interviews with specific individuals. Since 2003, annual HIV surveillance has been conducted with a stratified sample of non-resident household members living in households outside the DSA (Tanser *et al*., 2008). The eligibility list for the sample is drawn up on a date shortly before the start of data collection and includes all females aged 15–49 years and all males aged 15–54 years who were recorded as non-resident household members in the ACDIS as of that date. Strata are defined by sex and pattern of return visits to their household in the DSA. The sample also includes all non-resident individuals who had a negative HIV test result in HIV surveillance in the 2 years preceding the survey but had not been randomly selected into the sample.

#### The Non-Residents Living Arrangements Survey

In 2009, the NRLA survey was included as an additional module to the questionnaire administered to the sample included in non-residents HIV surveillance. The purpose of this survey was to investigate migrants' partnership patterns and determine the aspects of migration that contribute to risky sexual behaviour (McGrath *et al*., 2008). The data collected in this cross-sectional module included the following:

- The current employment status of non-residents.
- Non-residents' presence pattern in their origin and destination households over the 6 months preceding their survey interview.
- Membership details for non-residents' destination household, including each household member's relationship to the non-resident and whether the non-resident considered them to also be a member of their DSA household.
- Non-residents' sexual behaviour and living arrangements with partners in the 12 months preceding the survey in both the destination and origin household.

The information collected on members of non-residents' destination households also makes the NRLA survey a suitable data source for examining the living arrangements of migrants’ children.

#### Sampling strategy and response rate

The eligibility list for the non-residents HIV surveillance (and the NRLA survey) included 2841 individuals and was constructed based on information from the previous ACDIS household visit prior to 22/12/2008, therefore, could have been collected up to 6 months beforehand. By the scheduled interview day in 2009, 14 individuals had died, 1233 were uncontactable or had out-migrated from the address provided at the time of their migration from of the DSA, and 495 were unable to complete the survey for other reasons. An additional 217 were found to be resident in the DSA, so were not eligible to complete the survey. Amongst individuals who remained eligible to complete the survey, 63% responded, providing a dataset containing information on 560 individuals. The reduced size of the dataset is a limitation of this analysis and relates to the inherent difficulties of capturing data on mobile populations. Furthermore, the smaller sample size compared with the sample size if all individuals on the eligibility list had participated, may lead to bias in the results if certain groups were more likely to respond than other groups. However, a comparison of the age, sex, and residential history of participants and non-participants did not reveal statistically significant differences between participants and non-participants (Table 1). Furthermore, probability weights were calculated and applied throughout the analyses to account for the probability of selection and response. In this paper, we refer to the non-resident respondents as ‘migrants’. ‘Destination household’ is used to refer to the migrant's household outside the DSA where the survey interview was conducted, and ‘origin household’ is used to refer to the household where they are reported to be a member in the DSA.

**Table 1:** Characteristics of non-participants and participants.

	Non-participant		Participant		Total		*p*-value^a^
	*N*	Row %	*N*	Row %	*N*	Row %	
Sex							0.963
Female	1138	80	280	20	1418	100	
Male	1143	80	280	20	1423	100	
Age (years)							0.387
<20	294	81	68	19	362	100	
20–24	629	79	169	21	798	100	
25–29	477	80	118	20	595	100	
30–34	290	81	68	19	358	100	
35–39	247	81	57	19	304	100	
40–44	165	85	28	15	193	100	
45–49	105	75	35	25	140	100	
50–54	74	81	17	19	91	100	
Residential history in the DSA							0.269
Never a resident in the DSA since 1/1/2000	520	79	140	21	660	100	
At least one residency episode in the DSA since 1/1/2000	1761	81	420	19	2181	100	
Total	2281	80	560	20	2841	100	

DSA, demographic surveillance area.

a Pearson chi-squared statistic. Testing for differences between non-participants and participants.

#### Identifying and characterising migrant parents

The survey included a question that asked whether the respondent considered each member of their destination household to also be a member of their origin household in the DSA. If a migrant indicated that an individual was a member of both their households, their information in the two data sources were linked based on the individual's date of birth, sex, name, and parents' vital status. Migrant respondents were identified as parents if they reported at least one child as a member of their destination household, and/or were registered as the parent of at least one different living child in the ACDIS database on their survey interview day; 233 [34% (weighted %)] migrants were identified to be parents, linked to a sample of 458 children. Migrant parents are more likely to be female, older, employed, and in a relationship than non-parent migrants (Table 2).

**Table 2:** Characteristics of parent and non-parent migrant respondents^a^.

	Parents %	Non-parents %	Total %	*p*-value^b^
Sex				0.0003
Male	32	58	48	
Female	68	42	52	
Age (years)				
<25	18	55	42	<0.001
25–34	36	33	34	
35–44	27	9	15	
45+	20	4	9	
Length of migration episode (years)				0.068
<3	26	39	35	
3–7	45	42	43	
8+	30	18	22	
Partnership arrangement				<0.001
No partner in either household	67	94	85	
Partner (member of destination household only)	12	3	6	
Partner (member of both households)	9	2	4	
Partner (member of origin household only)	13	1	5	
Employment status				0.001
Employed (full-time or part-time)	77	50	59	
Student/training^c^	15	39	31	
Unemployed	8	12	10	

DSA, demographic surveillance area.

a Weighted column percentages based on 560 cases. Percentages may not sum to 100 because of rounding.

b Rao and Scott (1984) second-order correction to the Pearson chi-squared statistic (see also StataCorp (2009) p.122). Testing for differences between parents and non-parents.

c Answer given in response to the question ‘If currently unemployed, how do you spend the majority of your time during working hours?’

Table 3 presents the characteristics of migrant fathers and mothers. Migrant fathers are often long-term migrants with 47% having been away for 8 years or more. The majority of migrant fathers (95%) and migrant mothers (69%) are employed. In addition, 21% of migrant mothers are students or in training. There are differences in the pattern of social relationships that fathers and mothers have at their place of residence. Migrant fathers are more than twice as likely to be in a sexual relationship with a partner in either household as migrant mothers. Migrant fathers are most likely to have a partner in their origin household, whereas mothers are most likely to have a partner in their destination household. Over 95% of migrant fathers and mothers reported spending at least one night in their origin household in the preceding six months, suggesting that return visits continue to be an important way for migrants to remain connected to their rural households.

**Table 3:** Characteristics of migrant parents by sex^a^.

	Mothers %	Fathers %	Total %	*p*-value^b^
Age (years)				
<25	24	5	18	0.023
25–34	40	27	36	
35–44	22	36	26	
45+	14	31	20	
Length of migration episode (years)				0.017
<3	27	23	26	
3–7	51	30	45	
8+	22	47	30	
Partnership arrangement				0.037
Single	74	50	67	
Partner (member of destination household only)	13	9	12	
Partner (member of both households)	7	13	9	
Partner (member of origin household only)	6	27	13	
Employment status				<0.001
Employed (full or part-time)	69	95	77	
Student/training	21	0	14	
Unemployed	10	5	8	
Nights spent in origin household in 6 months preceding survey interview				0.70
None	1	2	1	
Less than 30 days	76	76	76	
30 days or more	24	22	23	

a Weighted column percentages based on 233 cases. Percentages may not sum to 100 due to rounding.

b Rao and Scott (1984) second-order correction to the Pearson chi-squared statistic. Testing for differences between mothers and fathers.

c HH is used to refer to household.

#### Linking data sources

Respondents were asked if they considered each member of their destination household to also be a member of their origin household. For household members considered members of both households, their data from the NRLA survey and the ACDIS were linked as far as possible. Initially, an automated search was conducted to find matches in the NRLA survey and the ACDIS data based on date of birth, sex, name, and parents' vital status. Secondly, a manual search was conducted to find highly plausible matches by examining all the available information on the migrant respondent's children available in the ACDIS on a case-by-case basis. This made it possible to match data for children with some missing or incomplete data, or where the data in the two sources varied slightly (e.g. if a date of birth was approximate or an abbreviated name was provided in one source). In total, it was possible to match data for 68% of migrants' children considered a member of both households. In a small number of cases (*n* = 8) it was possible to link records for a child who was a member of their parent's destination household but for whom their migrant parent had indicated they did not consider them a member of their origin household. In these analyses, only children for whom their migrant parent indicated during their interview that they were members of both households are considered members of both households.

### Using the NRLA survey data to examine the social and residential connections between left behind children and their migrant parents

This section applies the data from the NRLA survey linked to the ACDIS data to examine the residential arrangements of left behind children in the context of parental migration. The results document the social and residential connections between children and migrant parents and the migration histories of left behind children using descriptive statistics.

The results presented in Table 4 confirm that two decades after the restrictions on family migration were lifted, it remains uncommon for children to be included in the destination household of migrant parents. Only a very small group of children are members of their migrant parent's destination household only (5%; column A) and a slightly larger group are members of their migrant parent's origin and destination households (13%; column B). Over 99% of children who were members of their parent's destination household only (column A) or members of their parent's origin and destination households (column B) are resident members of the destination household, indicating that membership of the destination household is very closely linked to physical presence in the household. In a companion paper published in this issue (Bennett *et al*., 2014), we present a detailed analysis of the circumstances of children's inclusion in the parental destination household.

**Table 4:** Children's characteristics by their household memberships and residential locations in relation to migrant parent^a^.

	Destination HH (A) %	Origin & destination HH (B) %	Origin HH (resident) (C) %	Origin HH (non-resident)^c^ (D) %	Different HH in DSA (E) %	Total %	*p*-value^b^
Sex							0.038
Male	42	57	52	34	26	49	
Female	58	43	48	67	74	51	
Child's Age (yrs)							0.024
<5	40	40	17	26	39	24	
5–9	44	21	38	32	3	34	
10–17	16	39	44	42	58	42	
Born before start of parent's migration episode	38	56	82	60	77	72	0.0001
**% of children by HH memberships and residential location (Row %)**	**5**	**13**	**58**	**21**	**4**	**100**	

HH, household; DSA, demographic surveillance area.

a Weighted column percentages based on 458 cases. Percentages may not sum to 100 because of rounding.

b Rao and Scott (1984) second-order correction to the Pearson chi-squared statistic. Testing for differences by children's household memberships and residential locations.

c This group are residents in a household outside the DSA, which is not their migrant parent's destination household.

The majority of migrants' children (58%; column C) are resident members of their parent's origin household only. In migration literature, this group of children would typically be described as having been left behind by migrant parents. An additional 21% are members of their parent's origin household but are *non-resident* members (column D). This provides quantitative evidence that a significant proportion of migrants' children are not residing in their migrant parent's origin *or* destination household, but are residing in a different household outside the DSA. From the perspective of the ACDIS data only, it would only be possible to observe that the child is non-resident and the parent is non-resident, but not that they are not residing together in their destination.

The finding that a significant proportion of children are non-resident members of their parent's origin household is in contrast to the finding noted earlier that over 99% of children who are members of their parent's destination household are resident members. These differences in patterns of membership and residency in origin and destination households ties in with the popular notion in African migration literature that migrants have a ‘rural home’ and an ‘urban dwelling’ (Datta, 1995). The origin household constitutes an inclusive family base, whereas the destination household constitutes a smaller group of linked individuals who live together elsewhere.

A further 4% of children are members of a household in the DSA but not their parent's origin household (column E). This group are likely to be underrepresented in these analyses, as parent–child relationships will only be recorded in the demographic surveillance system if the parent and child have ever shared a household membership. However, the group highlights the fact that migrant parents may have more children than is possible to see from examining their origin and destination households only.

The individual characteristics of children left behind differ by their social and residential arrangements. For example, children who are non-resident members of their migrant parent's origin household (column D) or are not members of their migrant parent's origin or destination household (column E) are more likely to be younger than 5 years (*p* = 0.053) than children who are resident members of their migrant parent's origin household (column C), which may relate to accessing alternative caregivers in the absence of their migrant parent.

A key benefit of using surveillance data from the ACDIS in parallel to the NRLA survey data is the detailed longitudinal data, which have been collected prospectively since 2000 on living arrangements of children and parents available in the surveillance data. Table 5 presents findings on the extent to which the migration and residential history of the three groups of left behind children identified earlier (columns C–E in Table 4) are associated with those of their migrant parent. The results show the majority (75%) of left behind children were recorded in the ACDIS to have migrated at least once.^1^ This level of mobility is significantly higher than other children in the study area: 49% of all children resident in the DSA on 1/1/2009 had been recorded in the ACDIS to have moved at least once.^2^ Older left behind children (5 years and older) are significantly more likely to have migrated at least once than younger left behind children (82% compared with 48%, *p* < 0.001, result not shown).

**Table 5:** Residential and migration history for ‘left behind’ children by social and residential connection to migrant parent's origin household a.

	Origin HH (resident) %	Origin HH (non-resident) %	Different HH in DSA %	Total %	*p*-value^b^
1+ migration^c^	73	80	67	75	*0*.*56*
Periods of co-residency with migrant parent in DSA since 1/1/2000 for children born before the start of their parent's migration^d^:	*0*.*063*				
1+	63	56	16	59	
0	37	44	84	41	
Of children born before the start of their parent's migration who have had 0 periods of co-residency with migrant parent in DSA since 1/1/2000^e^:					*0*.*24*
Migrant parent never resident in DSA since 1/1/2000	88	76	67	84	

HH, household; DSA, demographic surveillance area.

a Percentages may not sum to 100 because of rounding.

b Rao and Scott (1984) second-order correction to the Pearson chi-squared statistic. Testing for differences by children's household memberships and residential locations.

c Weighted column percentages based on 354 cases.

d Weighted column percentages based on 266 cases.

e Weighted column percentages based on 171 cases.

Many children born before the start of their parent's migration had never been co-resident with their migrant parent in the DSA (40%), particularly those who are a member of neither parental household. Family members other than biological parents, most commonly grandmothers and aunts, often act as primary caregivers to children, frequently for extended periods. For the majority of children who have never been co-resident with their migrant parent, their parent migrated before the start of surveillance. For the others, their migrant parent had been resident with a household in the DSA in the child's lifetime, but never with the child. Children who do not live with their migrant parent prior to migration may have a different experience of parental migration to those that were co-resident with a parent, especially if the parent was the child's primary caregiver, however, are rarely captured in studies of migrants' children.

The proportion of left behind children who have never been co-resident with their migrant parent is higher for children with migrant fathers, expected given the lower rates of father–child co-residency in South Africa (results not shown). However, the pattern shown in Table 5, whereby left behind children are most likely to have shared a period of residency with their migrant parent if they are resident member of their migrant parent's origin household, and least likely to have if they are a member of neither parental household, is also true amongst children with migrant mothers only and amongst children with migrant fathers only (results not shown).

## Discussion

In South Africa, sociologists, anthropologists, and demographers frequently highlight the fluidity and complexity of family arrangements and identify limitations of survey-type instruments in adequately representing and modelling contemporary residential and social arrangements (Russell, 2003; Hosegood *et al*., 2005; Amoateng & Richter, 2007). The collection and presentation of data from multiple households in different locations is very challenging in all studies and magnified given the size of the surveillance population. In South Africa, the first wave of the Cape Area Panel Study collected information on biological children of household members not residing in the household and the National Income Dynamics Study collects data on the province and type of accommodation non-resident household members reside in. However, these data sources do not include information on the composition of the households non-residents live in. This study shows that it is possible to collect information that allows investigation of family migration, and in particular, the arrangements of children left behind by migrant parents. Improvements in the availability of data on dispersed families in South Africa, particularly in the context of high levels of migration and comparatively low rates of parental co-residence, have been advocated as important to studies of other aspects of family demography and health including father involvement and child care (Madhavan *et al*., 2008; Sherr *et al*., 2008; Hosegood & Madhavan, 2010).

The linked data from the surveillance and the NRLA survey has permitted insights into the arrangements of left behind children in South Africa that would not have been possible from a survey or surveillance alone. These include (i) the identification of a significant group of left behind children who were not resident in their migrant parent's origin household in the surveillance area and (ii) the finding that the majority of children not included in their parental destination household have migrated previously, and are significantly more mobile than the population of resident children in the surveillance area. Despite an acceptance in the substantive literature that migrants' children may reside with extended family, operationalising children left behind in empirical studies as resident members of the parental origin household has contributed to an association between left behind children and immobility (Kothari, 2002; Whitehead & Hashim, 2005). These results provide quantitative evidence for the need to pay greater attention to the dynamic nature of the residential arrangements of migrants' children in the context of South Africa and for a more inclusive conceptualisation of left behind children.

The approach used in the NRLA survey is one that could be repeated in many other settings given that there are over 30 demographic surveillance systems conducted in low and middle-income countries (Baiden *et al*., 2006). Several of these DSSs have adopted a definition of household membership, which includes resident *and* non-resident members, and can therefore be used to generate a sampling frame for conducting a representative nested sample survey of non-residents. One change to the approach used in the NRLA survey, which might usefully be considered in any future survey, is to pre-print information about the migrants' origin household as a means of guiding interviews with informants at the destination household and facilitating matching. During the development phase of the NRLA survey, the use of a pre-printed household roster with names, ages, and other identifying information to guide the interview and facilitate the matching of individuals in both origin and destination was considered. However, some staff were concerned that presenting this list to non-resident members might create the impression that data confidentiality was not being maintained. As this was the first attempt at establishing linkages with households outside the DSA, it was decided not to pilot the pre-printed list strategy to test whether participants were comfortable or not with its use. The considerable advantages of using such a list to facilitate the linkages more quickly and accurately would warrant exploration for feasibility in any future data collection activities with non-resident members.

The NRLA survey identified migrants' children who were members of the parental destination household and/or members of at least one household in the DSA. However, information was not available on children living in other households outside the DSA. Furthermore, the group of children who were not members of their parent's origin or destination household identified in these analyses are likely to be underrepresented, as parent–child relationships are only recorded in the ACDIS if the child and parent have ever shared household membership. For understanding the spatial distribution of children and migrant parents, one significant adaptation to the NRLA survey and existing household surveys would be to ask adult respondents about the living arrangements of *all* of their children. In addition, it would be valuable to include questions about the residential location of parent(s) of child members residing in households, which do not include their parent(s).

In summary, this paper makes a unique contribution to the literature on children and migration by describing the methodology for using data collected in migrants' origin and destination households for examining the social and residential connections between children and migrant parents. The empirical findings present new insights into the residential arrangements of left behind children in South Africa and highlight the need for careful measurement and conceptualisation of the circumstances of left behind children and further research on children's own migratory patterns.

## Footnotes

1 Since birth, start of demographic surveillance area household membership or the start of surveillance in 2000 (whichever was latest).

2 Since birth, start of demographic surveillance area household membership or the start of surveillance in 2000 (whichever was latest).
